# Global gene expression profiling of oral cavity cancers suggests molecular heterogeneity within anatomic subsites

**DOI:** 10.1186/1756-0500-1-113

**Published:** 2008-11-13

**Authors:** Patricia Severino, Adriana M Alvares, Pedro Michaluart, Oswaldo K Okamoto, Fabio D Nunes, Carlos A Moreira-Filho, Eloiza H Tajara, Head and Neck Genome Project GENCAPO

**Affiliations:** 1 Centro de Pesquisa Experimental, Instituto Israelita de Ensino e Pesquisa Albert Einstein, São Paulo, SP, Brazil; 2 Laboratório de Biologia Molecular, Hospital Heliópolis, São Paulo, SP, Brazil; 3 Departamento de Cirurgia de Cabeça e Pescoço do Hospital das Clínicas, Faculdade de Medicina da Universidade de São Paulo, São Paulo, SP, Brazil; 4 Departamento de Neurologia e Neurocirurgia, Universidade Federal de São Paulo, São Paulo, SP, Brazil; 5 Departamento de Estomatologia, Faculdade de Odontologia da Universidade de São Paulo, São Paulo, SP, Brazil; 6 Departamento de Pediatria, Faculdade de Medicina da Universidade de São Paulo, São Paulo, SP, Brazil; 7 Departamento de Biologia Molecular, Faculdade de Medicina de São José do Rio Preto, São José do Rio Preto, SP, Brazil; 8 Departamento de Genética e Biologia Evolutiva, Instituto de Biociências da Universidade de São Paulo, São Paulo, SP, Brazil

## Abstract

**Background:**

Oral squamous cell carcinoma (OSCC) is a frequent neoplasm, which is usually aggressive and has unpredictable biological behavior and unfavorable prognosis. The comprehension of the molecular basis of this variability should lead to the development of targeted therapies as well as to improvements in specificity and sensitivity of diagnosis.

**Results:**

Samples of primary OSCCs and their corresponding surgical margins were obtained from male patients during surgery and their gene expression profiles were screened using whole-genome microarray technology. Hierarchical clustering and Principal Components Analysis were used for data visualization and One-way Analysis of Variance was used to identify differentially expressed genes. Samples clustered mostly according to disease subsite, suggesting molecular heterogeneity within tumor stages. In order to corroborate our results, two publicly available datasets of microarray experiments were assessed. We found significant molecular differences between OSCC anatomic subsites concerning groups of genes presently or potentially important for drug development, including mRNA processing, cytoskeleton organization and biogenesis, metabolic process, cell cycle and apoptosis.

**Conclusion:**

Our results corroborate literature data on molecular heterogeneity of OSCCs. Differences between disease subsites and among samples belonging to the same TNM class highlight the importance of gene expression-based classification and challenge the development of targeted therapies.

## Background

Head and neck squamous cell carcinomas (HNSCC) rank among the top ten most common cancers worldwide, with increasing rates in certain areas of the world [1]. They can occur at different subsites, often invading more than one, each one with their own particular problems regarding management. In this study, we focused on oral squamous cell carcinoma (OSCC), a frequent neoplasm, which is usually aggressive and has unpredictable biological behavior and unfavorable prognosis. The accumulation of genetic alterations during oral tumorigenesis, leading to qualitative and quantitative changes in gene expression, is currently known but still largely unexplored [2,3]. Unlike estrogen receptor or HER2 in breast cancer, no biomarkers are currently available for HNSCC prognosis, which depends largely on the stage at presentation, with the most important prognostic factor being the presence of neck node metastases [4]. Improvements in specificity and sensitivity of diagnosis and in disease monitoring depend on the elucidation of the biological and molecular mechanisms underlying tumor development. In this context, large-scale transcriptome analysis may be used to assess patterns of gene expression, providing an opportunity to investigate the complex cascade of molecular events leading to tumor development and progression [5,6].

Two recent publications have examined the global gene expression profiles of OSCC, both addressing the importance of understanding the molecular complexity of this malignancy [7,8]. Unlike those authors, we did not focus on molecular differences between OSCC and normal mucosa, but we compared gene expression profiles of OSCC samples from different anatomic subsites. The biologic behavior of OSCC cannot be predicted and clinical reports suggest that molecular heterogeneity of anatomic subsites could play an important role in this scenario. For instance, cancer of the tongue seems to grow faster than other oral cavity cancers, with cervical metastases occurring more frequently in such cancers[9]. Moreover, significant differences between floor of the mouth and tongue cancers in response to combined surgery and radiotherapy have been reported [10].

In order to evaluate molecular differences between OSCC anatomic subsites, we investigated samples of primary tongue and floor of the mouth tumors, and their corresponding surgical margins, in respect to their gene expression profile by means of DNA microarrays.

## Methods

We selected samples of primary tongue and floor of the mouth tumors, and their corresponding surgical margins, from 9 male patients during surgery for squamous cell carcinomas. Samples consisted of either small tumors but already with evidences of metastasis (T2N+), or larger, non-metastatic samples (T3N0). Patients had a history of tobacco and alcohol abuse and no prior cancer treatment. A full description of the clinical data, including tumor stage is provided in Additional File 1. All participants provided written consent and the research protocol was approved by review boards of all involved institutions. Analysis of haematoxylin and eosin-stained sections indicated that each OSCC sample contained at least 70% tumor cells and the corresponding surgical margins were reported "tumor-free". Total RNA was extracted with TRIZOL Reagent (Invitrogen). Tissues adjacent to resected tumors were grouped into two pools of RNA for microarray analysis, corresponding to T2N+ and T3N0 samples, due to the limited amounts of tumor-free margin available in most cases.

Microarray experiments were carried out using CodeLink Whole Genome Bioarrays (GE Healthcare) and arrays were scanned on a GenePix 4000B Array Scanner (Axon Instruments), according to the recommended scanning procedures and settings. The data were treated with CodeLink feature extraction software v.4.0. A normalized signal for each transcript was obtained through quantile normalization [11]. The array design and raw data files are available at the Gene Expression Omnibus database (GEO) under the accession number GSE9792. For global gene expression visualization, we used hierarchical clustering and Principal Components Analysis (PCA). Hierarchical clustering was performed using the Euclidean distance and the average linkage algorithm. One-way ANOVA was used to identify significant differences in the dataset. All the above-mentioned analyses were generated using Partek^® ^software version 6.3 Copyright^© ^2007 Partek Inc., St. Louis, MO, USA. Functional annotations of differentially expressed genes were performed using Database for Annotation, Visualization and Integrated Discovery [12] using the parameters Gene Ontology (GO) Molecular Process term level 5 and KEGG Pathways (Kyoto Encyclopedia of Genes and Genomes)[13].

In order to corroborate our findings, two publicly available microarray datasets on OSCC were used in addition to our data (GEO accession number GSE3524 and ArrayExpress accession number TABM302). Both experiments were carried out using Affymetrix HG-U133 GeneChips; GSE3524 corresponds to the profiling of 7 tongue and 9 floor of the mouth samples, while TABM302 corresponds to the profiling of 18 tongue samples and 10 samples reported as oral cavity. Statistical analysis and functional annotation of the differentially expressed genes followed the procedures previously described for our dataset (Additional Files 2 and 3).

## Results and Discussion

**Figure 1:**
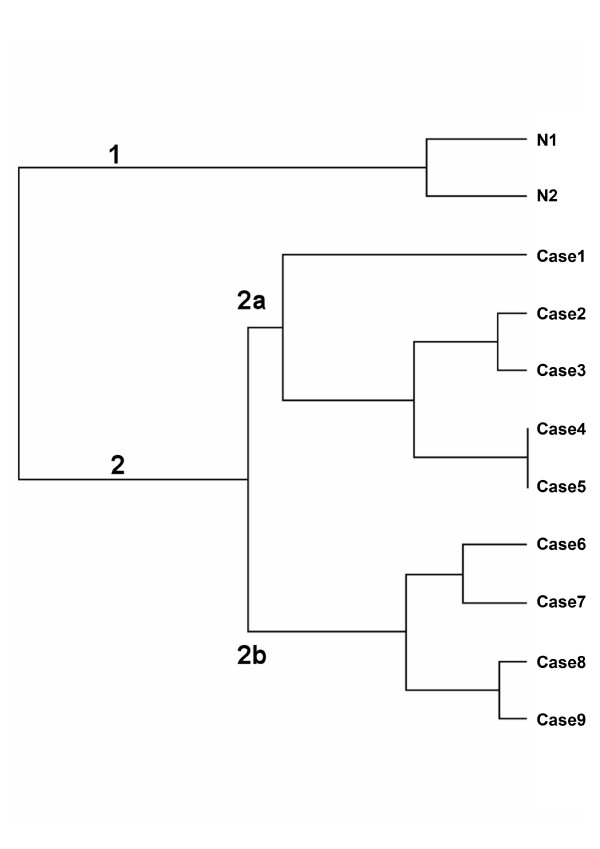
**Hierarchical clustering**. The analysis was performed using the Euclidean distance and the average linkage algorithm. N1: Surgical margins from T2N+ samples; N2: Surgical margins from T3N0 samples. Case1–9: Tumor samples described in Additional File 1.

**Figure 2:**
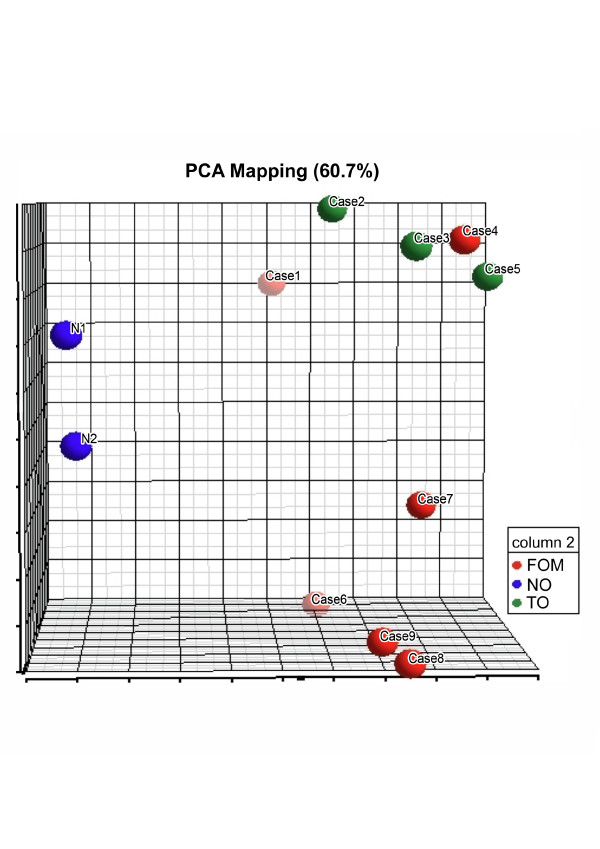
**Three-dimensional Principal Components Analysis**. FOM: Floor of the mouth, NO: Surgical margins (N1: Surgical margins from T2N+ samples; N2: Surgical margins from T3N0), TO: Tongue.

By analyzing the dendogram resulting from hierarchical clustering and the PCA, we observed that OSCC samples did not group according to their pathological stages (Figures 1 and 2, respectively). In agreement with previous reports [14,15], two major clusters reflected differences in global gene expression between non-tumoral tissues (cluster 1) and tumor samples (cluster 2). Tumor samples clustered mostly, but not exclusively, according to disease anatomic subsites. This result suggests molecular heterogeneity within TNM classes and corroborates previous data published by Ziober et al. [15] and Chung et al[16]. In the later study, there were no consistent differences in gene expression among HNSCC subsites, but samples from oral cavity seemed more heterogeneous than samples from other sites when expression patterns were evaluated. Few studies have aimed to understand the molecular background of OSCC. In the context of molecular target therapies, drugs are designed to interact with specific molecules present in certain types or subtypes of tumors. Due to the heterogeneity within HNSCC, a thoroughly comprehension of the molecular characteristics underlying its subsites is needed before efficient therapy can be achieved.

### Differential gene expression profiles

Using hierarchical clustering and PCA, two main groups of tumor samples (2a and 2b) were observed (Figure 1). Each group contained, mostly, either tongue or floor of the mouth samples. Differentially expressed genes between the two clusters were identified using ANOVA (Additional File 4). These genes (total number 1579) were functionally annotated using Gene Ontology (GO) and KEGG terms in an attempt to understand major molecular differences between the groups (Tables 1 and 2). Due to our small sample number, our results were compared with two additional OSCC microarray datasets, GSE3524 and TABM302. Despite broad differences in experimental design, probe sets, and possibly genetic background, some findings were consistent and are pointed out below.

**Table 1:** GO terms containing differentially expressed genes between OSCC subsite-related clusters

**GO Term**	**p Value**	**Gene Symbol**
GO:0006412:translation	1.55E-07	C15ORF15, CEBPG, DARS2, DDX1, DENR, EEF1A1, EIF1, EIF1AY, EIF2B1, EIF2B3, EIF2C4, EIF3S7, EIF4A1, EIF4EBP1, ETF1, GSPT1, HARSL, HBS1L, IGF2BP2, JTV1, LARS, LOC151507, MRPL1, MRPL10, MRPL20, MRPL22, MRPL35, MRPL37, MRPL45, MRPL9, MRPS15, MRPS16, MRPS7, MTIF2, NARS2, NFKB1, PAIP2, PDF, PET112L, PRG3, RPL13, RPL18A, RPL19, RPL26, RPL26L1, RPL27, RPL35A, RPL36A, RPL4, RPL6, RPL7, RPL7A, RPLP1, RPS14, RPS15A, RPS2, RPS24, RPS26, RPS26E, RPS26L, RPS27, RPS27A, RPS3, RPS3A, RPS7, SAMD4A, SARS, SECISBP2, SLA/LP, SRP9, STAT5B, TNIP1, TRIP11, TSFM, TUFM, WBSCR1, ZFP36L1
GO:0000087:M phase of mitotic cell cycle	5.53E-06	ANAPC11, APRIN, AURKB, BIRC5, C20ORF172, CCNB1, CCNK, CDC2L5, CLASP2, CSPG6, EML4, FAM33A, GTSE1, KIF2C, KNTC1, LUZP5, MAD2L1, MPHOSPH6, NUDC, PAM, PB1, PRMT5, RAD21, RAN, RUVBL1, SMC4L1, SPAG5, SPBC25, STAG1, SUGT1, TARDBP, TTN, TXNL4B, ZWINT
GO:0007067:mitosis	1.21E-05	ANAPC11, APRIN, AURKB, BIRC5, C20ORF172, CCNB1, CCNK, CDC2L5, CLASP2, CSPG6, EML4, FAM33A, GTSE1, KIF2C, KNTC1, LUZP5, MAD2L1, NUDC, PAM, PB1, PRMT5, RAD21, RAN, RUVBL1, SMC4L1, SPAG5, SPBC25, STAG1, SUGT1, TARDBP, TTN, TXNL4B, ZWINT
GO:0006325:establishment and/or maintenance of chromatin architecture	1.37E-05	ARID1B, BAZ1B, C11ORF30, CBX3, CDYL, CEBPG, CHAF1A, CHD2, CHD9, EHMT2, EPC1, FBXL11, GCN5L2, H2AFX, H2AFZ, HDAC4, HDAC6, HIST1H3A, HIST1H3B, HIST1H3C, HIST1H3D, HIST1H3E, HIST1H3F, HIST1H3G, HIST1H3H, HIST1H3I, HIST1H3J, HIST3H3, HMGB2, HP1BP3, JARID1B, JMJD1C, JMJD2C, MBD3, MCM2, MLL3, NAP1L1, NCOR1, NSD1, PB1, PRMT5, RBBP4, RBL1, RERE, RUVBL1, SAS10, SET, SMARCB1, SMARCC1, WHSC1
GO:0000279:M phase	2.05E-05	ANAPC11, APRIN, AURKB, BIRC5, C20ORF172, C8ORF1, CCNB1, CCNK, CDC2L5, CLASP2, CSPG6, CTA-250D10.11, EML4, FAM33A, GTSE1, H2AFX, KIF2C, KNTC1, LUZP5, MAD2L1, MPHOSPH6, NUDC, PAM, PB1, PRMT5, RAD21, RAN, REC8L1, RUVBL1, SMC4L1, SPAG5, SPBC25, STAG1, SUGT1, TARDBP, TTN, TXNL4B, ZWINT
GO:0006323:DNA packaging	2.14E-05	ARID1B, BAZ1B, C11ORF30, CBX3, CDYL, CEBPG, CHAF1A, CHD2, CHD9, EHMT2, EPC1, FBXL11, GCN5L2, H2AFX, H2AFZ, HDAC4, HDAC6, HIST1H3A, HIST1H3B, HIST1H3C, HIST1H3D, HIST1H3E, HIST1H3F, HIST1H3G, HIST1H3H, HIST1H3I, HIST1H3J, HIST3H3, HMGB2, HP1BP3, JARID1B, JMJD1C, JMJD2C, MBD3, MCM2, MLL3, NAP1L1, NCOR1, NSD1, PB1, PRMT5, RBBP4, RBL1, RERE, RUVBL1, SAS10, SET, SMARCB1, SMARCC1, WHSC1
GO:0009260:ribonucleotide biosynthetic process	2.12E-04	ADK, ADSL, ATP5B, ATP5C1, ATP5F1, ATP5G3, ATP5H, ATP5L, ATP5O, ATP6V1C1, CTPS, IMPDH1, NME2, PAICS, PAPSS1, PAX8, UMPS
GO:0006281:DNA repair	2.87E-04	APEX1, ATRX, BLM, C11ORF30, C2ORF13, C8ORF36, CEBPG, CHAF1A, CIB1, CSNK1D, CSPG6, ERCC8, FANCC, FEN1, FLJ40869, H2AFX, HMGB2, LIG1, MBD4, MDC1, MGMT, MLH1, MSH3, NUDT1, POLE2, RAD18, RAD21, RBX1, REV1L, RPA3, SMC5L1, SOD2, XRCC5
GO:0006396:RNA processing	8.86E-04	AQR, BCAS2, C14ORF172, C1ORF19, CROP, CSTF2T, DDX1, DDX46, DDX5, DHX35, DICER1, ERN2, EXOSC1, EXOSC3, GEMIN5, HNRPA2B1, HNRPD, HSA9761, LOC113179, LSM3, MAGOH, NHP2L1, NOL5A, PABPN1, PDCD11, POP4, PPIL3, PRPF3, PRPF38B, RPS14, SARS, SAS10, SF3B14, SF3B5, SLBP, SMN1, SMN2, SNRPA1, SNRPF, SR140, SRRM2, TARDBP, TXNL4B, U2AF1, UTP11L, UTP15, ZCRB1, ZNF265
GO:0016071:mRNA metabolic process	9.55E-04	AQR, C1ORF19, CSTF2T, DCP1A, DDX1, DDX46, DDX5, DHX35, ERN2, GEMIN5, GSPT1, HNRPA2B1, HNRPD, LSM3, LSMD1, MAGOH, NHP2L1, PABPN1, PPIL3, PRPF3, PRPF38B, SF3B14, SF3B5, SLBP, SMG7, SMN1, SMN2, SNRPA1, SNRPF, SRRM2, TARDBP, TXNL4B, U2AF1, ZCRB1, ZFP36L1, ZNF265

**Table 2:** Major KEGG pathways containing genes differentially expressed between OSCC subsite-related clusters

**KEGG Term**	**p Value**	**Gene Symbol**
hsa03050:Proteasome	4.04E-03	PSMA1, PSMA2, PSMA4, PSMA5, PSMB2, PSMB6, PSMD12
hsa04350:TGF-beta signaling pathway	6.29E-02	ACVR2A, BMP7, CHRD, E2F4, PPP2R2B, RBL1, RBX1, SKP1A, SMAD4, SMURF2, TGFBR2, THBS3
hsa03010:Ribosome	2.80E-04	C15ORF15, MRPS7, RPL13, RPL18A, RPL19, RPL26, RPL27, RPL35A, RPL36A, RPL6, RPL7, RPS15A, RPS2, RPS24, RPS26, RPS26E, RPS26L, RPS27, RPS3, RPS3A, RPS7
hsa05210:Colorectal cancer	8.24E-03	AXIN2, BAX, BIRC5, CASP9, FZD8, HRAS, IGF1R, KRAS, MAPK9, MLH1, MSH3, NRAS, RALGDS, SMAD4, SOS1, TGFBR2
hsa00020:Citrate cycle (TCA cycle)	1.96E-02	ACLY, IDH3A, IDH3B, MDH2, SDHA, SDHB, SDHD
hsa01030:Glycan structures – biosynthesis 1	2.79E-02	ALG14, ALG9, B4GALT5, CHST11, CSS3, EXT1, FUT8, GALNACT-2, GALNT1, GALNTL4, HS3ST2, HS3ST3B1, MGAT5, OGT, ST3GAL1, XYLT1
hsa04110:Cell cycle	8.28E-03	ANAPC11, CCNB1, CDK4, DBF4, MAD2L1, MCM2, MCM3, MCM4, MCM5, MCM7, PTTG2, RBL1, RBX1, SKP1A, SMAD4, YWHAE, YWHAQ
hsa00190:Oxidative phosphorylation	2.50E-10	ATP5B, ATP5C1, ATP5F1, ATP5G3, ATP5H, ATP5L, ATP5O, ATP6V1C1, COX17, COX5A, COX6A1, COX6B1, COX7A2, COX7B, COX7C, CYC1, NDUFA12, NDUFA7, NDUFAB1, NDUFB10, NDUFB8, NDUFB9, NDUFS1, NDUFS4, NDUFS5, NDUFV2, PPA2, RP3-405J24.3, SDHA, SDHB, SDHD, UQCRB, UQCRFS1, UQCRH
hsa04540:Gap junction	8.56E-02	ADCY2, ADCY5, ADCY6, CSNK1D, GNAQ, GUCY1A3, HRAS, K-ALPHA-1, KRAS, NRAS, PDGFD, PRKG1, SOS1, TUBA6, TUBB6

Extensive differences in gene expression between clusters 2a and 2b were observed in respect to translation and mitosis-related GO terms (GO:0006412:translation, GO:0000087:M phase of mitotic cell cycle; GO:0007067:mitosis; GO:0000279:M phase). Translation initiation is regulated in response to mitogenic stimulation, thus coupled with cell cycle progression and cell growth. Several alterations in translational control occur in cancer, but there is still much to be discovered about their role in cancer development and progression [17]. Changes in the expression or availability of components of the translational machinery can lead to global changes, such as an increase in the overall rate of protein synthesis and translational activation of the mRNA molecules involved in cell growth and proliferation. In agreement, differences between the two clusters were enriched in GO terms Ribonucleotide Biosynthetic Process, RNA Processing and mRNA Metabolic Process as well as in KEGG Proteasome, Cell Cycle, TGFB Signaling and Ribosome Pathways. Components of the translation machinery and related pathways represent good targets for cancer therapy [18,19]. The differential expression of a number of genes involved in such processes suggests that the efficacy of oral cancer therapy should vary depending on the tumor subsite. Molecular mechanisms leading to differences in gene expression (i.e variations in mRNA sequences, changes in the availability of the translational machinery components, and activation of translation through abnormally activated signal transduction pathways) deserve to be investigated.

Differences in the expression of cell cycle progression-related genes were also observed in both GEO (GSE3524) and ArrayExpress (TABM302) datasets. In respect to the former, several genes associated with the KEGG MAPK Signaling Pathway presented differential expression when tongue samples were compared with floor of the mouth samples. Similar results were observed for GO terms concerning transcriptional regulation and apoptosis (Additional File 5). In agreement with our results, TABM302 dataset showed differential expression in genes associated with the GO terms Maintenance of Chromatin Architecture and DNA Packaging, both essential in cell cycle-related processes, and with the KEGG Ribosome Pathway (Additional File 6). Proliferation and cell death are also regulated by genes associated with the GO terms Small GTPase Mediated Signal Transduction and Regulation of Ras Protein Signal Transduction, both represented several times in the list of differentially expressed genes of the TABM302 dataset.

With respect to cell cycle and apoptosis, we also observed the over-expression in cluster 2b of three genes involved in the regulation of p27 phosphorylation during cell cycle progression (CKS1B, RBX1 and SKP1A) and genes from the MCM complex (minichromosomal maintenance genes), including MCM7 (Additional File 4). MCM7 protein is a marker for proliferation and it is upregulated in different tumors including neuroblastoma, prostate, cervical and hypopharyngeal carcinomas [20]. SKP1A, MCM3, MCM6 and MCM7 were also differentially expressed in TABM302 dataset (Additional File 3), however, their over-expression was observed in tongue samples.

Widespread differences were also observed in oxidative phosphorylation pathways (Tables 2 and 3). Genes belonging to KEGG Oxidative Phosphorylation and Citrate Cycle (TCA cycle) Pathways were over-expressed in cluster 2b. Oxidative phosphorylation of ADP to ATP accompanies the oxidation of a metabolite through the operation of the respiratory chain. According to Warburg [21], alterations of respiratory machinery should result in compensatory increase in glycolytic ATP production and lead to carcinogenesis. In fact, malignant cells meet their energy (ATP) needs by glycolysis rather than through oxydative phosphorylation, probably an adaptation to hypoxia that develops as tumor grows [22]. In accordance with our results, some authors have recently found a bioenergetic signature in several human tumors [23]. Our observations suggest that distinct OSCC subsites differ on their glycolytic phenotype, a matter that deserves further investigation and may impact tumor progression management as well as therapy outcome.

**Table 3:** KEGG enriched terms containing genes over-expressed in cluster 2b

**KEGG Term**	**p Value**	**Gene Symbol**
hsa00190:Oxidative phosphorylation	6.84E-16	ATP5B, ATP5C1, ATP5F1, ATP5G3, ATP5H, ATP5L, ATP5O, COX17, COX5A, COX6A1, COX6B1, COX7A2, COX7B, COX7C, CYC1, NDUFA12, NDUFA7, NDUFAB1, NDUFB10, NDUFB8, NDUFB9, NDUFS1, NDUFS4, NDUFS5, NDUFV2, PPA2, RP3-405J24.3, SDHA, SDHB, SDHD, UQCRB, UQCRFS1, UQCRH
hsa03050:Proteasome	2.58E-04	PSMA1, PSMA2, PSMA4, PSMA5, PSMB2, PSMB6, PSMD12
hsa04110:Cell cycle	0.001169541	ANAPC11, CCNB1, CDK4, DBF4, MAD2L1, MCM2, MCM3, MCM5, MCM7, PTTG2, RBX1, SKP1A, YWHAE, YWHAQ
hsa00020:Citrate cycle (TCA cycle)	0.001533882	ACLY, IDH3A, IDH3B, MDH2, SDHA, SDHB, SDHD
hsa03010:Ribosome	9.30E-07	C15ORF15, MRPS7, RPL13, RPL18A, RPL19, RPL26, RPL27, RPL35A, RPL6, RPL7, RPS15A, RPS2, RPS24, RPS26, RPS26E, RPS26L, RPS27, RPS3, RPS3A, RPS7

Genes related to KEGG Oxidative Phosphorylation Pathway also showed differences in expression in GSE3524 dataset when subsites were compared; the same was observed for genes related to the GO term Organelle ATP Synthesis Coupled Electron Transport (Additional File 5). The expression of several cytochrome oxidases and ATPases was also distinct between subsites in TABM302 (Additional File 3).

KEGG Pathway analysis revealed genes over-expressed in cluster 2a which are associated with gap junctions and adherens junctions, as well as cytoskeleton organization and biogenesis (Table 4), including ACTN, DOCK1, GSN, ITG, LIMK, RAS, SOS, SSH and TIAM1. In agreement with our data, genes related to KEGG Regulation of Actin Cytoskeleton and Adherens Junction Pathways were also found differentially expressed in TABM302 (Additional File 6). Cancer therapy has focused on such pathways. For example, microtubule-targeted drugs interfere with the ability of the cancer cells to divide and multiply by disrupting microtubules [24]. Gene-silencing may reduce expression of proteins involved in regulating the actin cytoskeleton, as LIMK, rendering cells more responsive to anticancer agents [25]. Therefore, molecular differences between OSCC subsites may affect sensitivity to chemotherapy.

**Table 4:** KEGG enriched terms containing genes over-expressed in cluster 2a

**KEGG Term**	**p Value**	**Gene Symbol**
hsa04510:Focal adhesion	0.075632796	ACTN3, DOCK1, FLNB, HRAS, IGF1R, ITGA11, KRAS, NRAS, PARVG, PDGFD, PTK2, SOS1, THBS3
hsa04810:Regulation of actin cytoskeleton	0.026522502	ACTN3, CYFIP2, DOCK1, GSN, HRAS, ITGA11, KRAS, LIMK2, NRAS, PTK2, RDX, RRAS2, SOS1, SSH2, TIAM1
hsa04540:Gap junction	0.007365075	ADCY2, ADCY5, ADCY6, GNAQ, GUCY1A3, HRAS, KRAS, NRAS, PDGFD, PRKG1, SOS1
hsa04520:Adherens junction	0.071052621	ACTN3, IGF1R, NLK, PTPRF, SMAD4, TGFBR2
hsa04350:TGF-beta signaling pathway	0.017990954	ACVR2A, CHRD, PPP2R2B, RBL1, SMAD4, SMURF2, TGFBR2, THBS3
hsa05210:Colorectal cancer	0.013445912	AXIN2, BAX, HRAS, IGF1R, KRAS, MSH3, NRAS, SMAD4, SOS1, TGFBR2
hsa01030:Glycan structures – biosynthesis 1	5.16E-05	B4GALT5, CHST11, CSS3, EXT1, FUT8, GALNACT-2, GALNT1, GALNTL4, HS3ST2, HS3ST3B1, MGAT5, OGT, ST3GAL1, XYLT1
hsa00532:Chondroitin sulfate biosynthesis	0.027092201	CHST11, CSS3, GALNACT-2, XYLT1
hsa00512:O-Glycan biosynthesis	0.014720589	B4GALT5, GALNT1, GALNTL4, OGT, ST3GAL1

In addition to the data presented above, several genes over-expressed in cluster 2b have been previously associated with oral cancer, such as GSTM1, IL8, MGMT, NFKB1 and NUDT1; as well as with pancreatic cancer (APEX1, CDK4, FANCC, GSTM1, MGMT and SOD2), lung cancer (APEX1, CASP9, GSTM1, HSPA8, IL8, LIG1, MBD4, MGMT, MLH1, NUDT1 and SOD2), colorectal cancer (APEX1, BLM, GSTM1, IL8, MGMT, MLH1, NFKB1, NUDT1, RFC1, SOD2 and UMPS) and bladder cancer (APEX1, GSTM1, IL8, LIG1, MGMT and SOD2) (Additional File 4). We believe the differential expression between anatomic subsites of genes known to be involved in tumorigenesis deserves further investigation.

## Conclusion

Gene expression profiling of lymph node positive and lymph node negative HNSCC has been addressed in the literature [14,15,26,27]. Besides the differences in study designs, the lack of consistency concerning HNSCC gene expression signatures could be due to gene expression variation related to subsites. This is a preliminary study and as such presents results on a small number of samples. However, the idea of molecular heterogeneity within HNSCC is not new. We corroborate previously reported literature data and we suggest that differences between anatomic OSCC subsites could impact drug response and should be considered during the development of targeted therapies.

## Competing interests

The authors declare that they have no competing interests.

## Authors' contributions

PS carried out hybridization experiments, data analysis and drafted the manuscript; AMA coordinated sample collection and carried out clinical data analysis; PM carried out clinical data analysis for sample selection; OKO helped with data analysis and the study design; FDM participated in the study design and carried out clinical data analysis; GENCAPO team members were responsible for sample collection, initial on-site sample processing, provided the pathological analysis of the cases, obtained the informed consent and discussed the findings. CAMF participated in the study design and coordination; EHT conceived the study, carried out data analysis and coordination. All authors read and approved the final manuscript.

## Supplementary Material

Additional File 1 **Clinicopathological features of patients in this study.** Click here for file

Additional File 2 **Differentially expressed genes between tongue and floor of the mouth samples from GEO dataset GSE3524 (p ≤ 0.05).** Click here for file

Additional File 3 **Differentially expressed genes between tongue and oral cavity samples from ArrayExpress dataset TABM302 (p ≤ 0.05).** Click here for file

Additional File 4 **Differentially expressed genes between OSCC clusters depicted by hierarchical clustering and PCA (p ≤ 0.05).** Click here for file

Additional File 5 **KEGG and GO terms containing differentially expressed genes between tongue and floor of the mouth samples from dataset GSE3524.** Click here for file

Additional File 6 **KEGG and GO terms containing differentially expressed genes between tongue and oral cavity samples from dataset TABM302.** Click here for file

## References

[B1] Shibuya K, Mathers CD, Boschi-Pinto C, Lopez AD, Murray CJ (2002). Global and regional estimates of cancer mortality and incidence by site: II. Results for the global burden of disease 2000. BMC Cancer.

[B2] Scully C, Field JK, Tanzawa H (2000). Genetic aberrations in oral or head and neck squamous cell carcinoma (SCCHN): 1. Carcinogen metabolism, DNA repair and cell cycle control. Oral Oncol.

[B3] Scully C, Field JK, Tanzawa H (2000). Genetic aberrations in oral or head and neck squamous cell carcinoma 3: clinico-pathological applications. Oral Oncol.

[B4] Greenberg JS, Fowler R, Gomez J, Mo V, Roberts D, El Naggar AK, Myers JN (2003). Extent of extracapsular spread: a critical prognosticator in oral tongue cancer. Cancer.

[B5] Huang E, Cheng SH, Dressman H, Pittman J, Tsou MH, Horng CF, Bild A, Iversen ES, Liao M, Chen CM (2003). Gene expression predictors of breast cancer outcomes. Lancet.

[B6] van 't Veer LJ, Dai H, Vijver MJ van de, He YD, Hart AA, Mao M, Peterse HL, Kooy K van der, Marton MJ, Witteveen AT (2002). Gene expression profiling predicts clinical outcome of breast cancer. Nature.

[B7] Sticht C, Freier K, Knopfle K, Flechtenmacher C, Pungs S, Hofele C, Hahn M, Joos S, Lichter P (2008). Activation of MAP kinase signaling through ERK5 but not ERK1 expression is associated with lymph node metastases in oral squamous cell carcinoma (OSCC). Neoplasia.

[B8] Kondoh N, Ishikawa T, Ohkura S, Arai M, Hada A, Yamazaki Y, Kitagawa Y, Shindoh M, Takahashi M, Ando T (2008). Gene expression signatures that classify the mode of invasion of primary oral squamous cell carcinomas. Mol Carcinog.

[B9] Ridge JA, Glisson BS, Horwitz EM, MN L (2007). Cancers of the Head and Neck Region. Textbook of cancer management: a multidisciplinary approach.

[B10] Zelefsky MJ, Harrison LB, Fass DE, Armstrong J, Spiro RH, Shah JP, Strong EW (1990). Postoperative radiotherapy for oral cavity cancers: impact of anatomic subsite on treatment outcome. Head Neck.

[B11] Bolstad BM, Irizarry RA, Astrand M, Speed TP (2003). A comparison of normalization methods for high density oligonucleotide array data based on variance and bias. Bioinformatics.

[B12] Dennis G, Sherman BT, Hosack DA, Yang J, Gao W, Lane HC, Lempicki RA (2003). DAVID: Database for Annotation, Visualization, and Integrated Discovery. Genome Biol.

[B13] The Cancer Genome Anatomy Project. http://cgap.nci.nih.gov/Pathways/.

[B14] Mendez E, Cheng C, Farwell DG, Ricks S, Agoff SN, Futran ND, Weymuller EA, Maronian NC, Zhao LP, Chen C (2002). Transcriptional expression profiles of oral squamous cell carcinomas. Cancer.

[B15] Ziober AF, Patel KR, Alawi F, Gimotty P, Weber RS, Feldman MM, Chalian AA, Weinstein GS, Hunt J, Ziober BL (2006). Identification of a gene signature for rapid screening of oral squamous cell carcinoma. Clin Cancer Res.

[B16] Chung CH, Parker JS, Karaca G, Wu J, Funkhouser WK, Moore D, Butterfoss D, Xiang D, Zanation A, Yin X (2004). Molecular classification of head and neck squamous cell carcinomas using patterns of gene expression. Cancer Cell.

[B17] Meric F, Hunt KK (2002). Translation initiation in cancer: a novel target for therapy. Mol Cancer Ther.

[B18] Hidalgo M, Rowinsky EK (2000). The rapamycin-sensitive signal transduction pathway as a target for cancer therapy. Oncogene.

[B19] Palakurthi SS, Fluckiger R, Aktas H, Changolkar AK, Shahsafaei A, Harneit S, Kilic E, Halperin JA (2000). Inhibition of translation initiation mediates the anticancer effect of the n-3 polyunsaturated fatty acid eicosapentaenoic acid. Cancer Res.

[B20] Honeycutt KA, Chen Z, Koster MI, Miers M, Nuchtern J, Hicks J, Roop DR, Shohet JM (2006). Deregulated minichromosomal maintenance protein MCM7 contributes to oncogene driven tumorigenesis. Oncogene.

[B21] Warburg O (1956). On the origin of cancer cells. Science.

[B22] Assaily W, Benchimol S (2006). Differential utilization of two ATP-generating pathways is regulated by p53. Cancer Cell.

[B23] Cuezva JM, Krajewska M, de Heredia ML, Krajewski S, Santamaria G, Kim H, Zapata JM, Marusawa H, Chamorro M, Reed JC (2002). The bioenergetic signature of cancer: a marker of tumor progression. Cancer Res.

[B24] Jordan MA (2002). Mechanism of action of antitumor drugs that interact with microtubules and tubulin. Curr Med Chem Anticancer Agents.

[B25] http://www.library.unsw.edu.au/~thesis/adt-NUN/public/adt-NUN20070830.132800/index.html.

[B26] Mendez E, Fan W, Choi P, Agoff SN, Whipple M, Farwell DG, Futran ND, Weymuller EA, Zhao LP, Chen C (2007). Tumor-specific genetic expression profile of metastatic oral squamous cell carcinoma. Head Neck.

[B27] Roepman P, Wessels LF, Kettelarij N, Kemmeren P, Miles AJ, Lijnzaad P, Tilanus MG, Koole R, Hordijk GJ, Vliet PC van der (2005). An expression profile for diagnosis of lymph node metastases from primary head and neck squamous cell carcinomas. Nat Genet.

