# Supplementary material for: Global gene expression profiling of oral cavity cancers suggests molecular heterogeneity within anatomic subsites
Source: BMC Res Notes. 2008 Nov 13;1:113. doi: 10.1186/1756-0500-1-113 (PMC2632665; doi:10.1186/1756-0500-1-113)
Supplement: Additional File 1 — Clinicopathological features of patients in this study. [file 1756-0500-1-113-S1.doc]

**Additional File 1.** Clinicopathological features of patients in this study.

| **Case** | **Site** | **Age (ys)** | **Pathologic stage** | **Histological differentiation** | **Lifetime smoking (yrs)** | **Pack-years** | **Lifetime alcohol consumption (yrs)** | **Alcohol consumption g/L/day^1^** | **Patient status** | **Survival months** | **Vascular infiltration** | **Lymphatic infiltration** | **Perineural invasion** |
| --- | --- | --- | --- | --- | --- | --- | --- | --- | --- | --- | --- | --- | --- |
| 1 | Floor of mouth | 46 | T2N2bM0 | Well | 33 | 49,50 | 10 | 1,12 | dd | 14 | No | No | No |
| 2 | Tongue | 43 | T3N0M0 | Moderate | 31 | 22,65 | 29 | 2291,78 | alive | 48 | Yes | Yes | No |
| 3 | Tongue | 47 | T3N0M0 | Moderate | 33 | 41,75 | 33 | 1485,88 | dd | 18 | No | No | Yes |
| 4 | Floor of mouth | 50 | T3N0M0 | Well | 30 | 18,20 | 30 | 316,81 | dc | 19 | No | Yes | Yes |
| 5 | Tongue | 29 | T2N2cM0 | Moderate | 6 | 4,80 | 5 | 18,77 | alive | 36 | No | Yes | Yes |
| 6 | Floor of mouth | 67 | T3N0M0 | Well | 52 | 72,50 | 49 | 459,55 | alive | 42 | No | No | No |
| 7 | Floor of mouth | 61 | T2N2bM0 | Moderate | 43 | 64,50 | 41 | 49,08 | dd | 8 | No | No | No |
| 8 | Floor of mouth | 62 | T3N0M0 | Moderate | 52 | 52,00 | 30 | 81,80 | alive | 48 | No | No | No |
| 9 | Floor of mouth | 56 | T2N2bM0 | Moderate | 40 | 50,00 | 40 | 192,86 | dd | 20 | No | No | No |

d = dead by disease; dc = dead by commorbidity. *Quantities correspond to beer and sugarcane distilled spirit (“*cachaça*”).

^1^grams/Liter/day
