# Supplementary material for: Global gene expression profiling of oral cavity cancers suggests molecular heterogeneity within anatomic subsites
Source: BMC Res Notes. 2008 Nov 13;1:113. doi: 10.1186/1756-0500-1-113 (PMC2632665; doi:10.1186/1756-0500-1-113)
Supplement: Additional File 5 — KEGG and GO terms containing differentially expressed genes between tongue and floor of the mouth samples from dataset GSE3524. [file 1756-0500-1-113-S5.doc]

Additional File 5: KEGG and GO terms containing differentially expressed genes between tongue and floor of the mouth samples from dataset GSE3524.

| **KEGG or GO term** | **p Value** | **Gene Symbol** |
| --- | --- | --- |
| hsa04010:MAPK signaling pathway | 1.52E-03 | ATF4, CACNA1G, CACNA1H, FGF22, FGF3, IL1A, IL1R2, JUND, MAP2K3, MAP3K5, MAPK14, MAPK7, MEF2C, NF1, NFATC4, PAK2, PDGFRA, PPM1A, PRKACA, RAP1A, RASA1, RASGRP2, TAOK3 |
| hsa00190:Oxidative phosphorylation | 1.09E-03 | ATP5C1, ATP5E, ATP5J2, ATP6V0A2, COX15, COX6A1, COX7A2, COX7B, NDUFA3, NDUFA4, NDUFA5, NDUFA6, NDUFB1, NDUFS8, PPA2 |
| GO:0042775~organelle ATP synthesis coupled electron transport | 1.00E-02 | COX15, NDUFA3, NDUFA4, NDUFA5, NDUFA6, NDUFB1, NDUFS8 |
| GO:0006915~apoptosis | 4.05E-02 | ADORA1, B4GALT1, BCAP29, BCAP31, CSF2, DAD1, ELMO2, FAF1, FIS1, GSK3B, IFT57, IL1A, KLRC4, LPAL2, MADD, MAP3K5, MBD4, MDM4, NF1, OSM, PAK2, PAWR, PDCD1, PPP2R1B, PRDX2, PRNP, PTH, RASA1, SERINC3, SOCS3, SQSTM1, TAX1BP1, TEGT, TM2D1 |
| GO:0045941~positive regulation of transcription | 4.45E-02 | ARHGEF11, ATF4, ATF7IP, CAMKK2, CD80, FOXH1, HNRPAB, MAP2K3, MED6, MEF2C, NCOA3, PAX8, RUNX1, SQSTM1, TCF1, TCF3, TCF4 |
| GO:0045893~positive regulation of transcription, DNA-dependent | 3.28E-02 | ARHGEF11, ATF4, ATF7IP, FOXH1, HNRPAB, MAP2K3, MED6, MEF2C, NCOA3, PAX8, RUNX1, SQSTM1, TCF1, TCF3, TCF4 |
