# Supplementary material for: Global gene expression profiling of oral cavity cancers suggests molecular heterogeneity within anatomic subsites
Source: BMC Res Notes. 2008 Nov 13;1:113. doi: 10.1186/1756-0500-1-113 (PMC2632665; doi:10.1186/1756-0500-1-113)
Supplement: Additional File 6 — KEGG and GO terms containing differentially expressed genes between tongue and oral cavity samples from dataset TABM302. [file 1756-0500-1-113-S6.doc]

Additional File 6: KEGG and GO terms containing differentially expressed genes between tongue and oral cavity samples from dataset TABM302.

| **KEGG or GO term** | **p Value** | **Gene Symbol** |
| --- | --- | --- |
| hsa04810:Regulation of actin cytoskeleton | 1.60E-02 | ACTB, ARAF, ARPC2, CDC42, CFL2, CHRM3, CYFIP2, FGF23, FGFR2, FGFR3, GNG12, IQGAP1, ITGA6, ITGAE, ITGAL, ITGB1, ITGB3, ITGB4, ITGB6, LIMK2, MAPK1, PAK3, PDGFA, PIK3CG, PIP5K1A, PPP1CC, PPP1R12A, RDX, SLC9A1, SOS2, TIAM1, VAV2, WASF2, WASL |
| hsa04520:Adherens junction | 3.90E-03 | ACP1, ACTB, CDC42, CSNK2A1, ERBB2, IGF1R, INSR, IQGAP1, MAPK1, MET, PARD3, PVRL4, SMAD3, SSX2IP, TJP1, WASF2, WASL |
| hsa03010:Ribosome | 8.05E-05 | LOC283340, LOC284393, LOC389342, LOC440589, RPL10A, RPL13, RPL13A, RPL14, RPL18, RPL24, RPL27A, RPL28, RPL29, RPL32, RPL7, RPS10, RPS11, RPS15A, RPS2, RPS20, RPS23, RPS28, RPS5, RPS9, RPSA |
| GO:0006323~DNA packaging | 2.28E-02 | ARID2, BAZ1B, BRD8, CHAF1A, CHD9, CPA4, EP400, FBXL10, GPX4, H2AFV, H2AFX, H2AFY2, H3F3B, HDAC4, HILS1, HIST1H4B, HIST1H4E, HIST1H4J, HIST1H4K, INOC1, JARID1B, JMJD1C, JMJD2D, MLL3, MYST1, MYST3, NAP1L1, NCOR1, PPARGC1A, PRM1, RB1, RCBTB1, RERE, RING1, SAFB, SETD7, SETDB2, SETMAR, SIRT4, SIRT7, SRCAP, TAF6L, TLK1, TSPYL5 |
| GO:0006325~establishment and/or maintenance of chromatin architecture | 2.66E-02 | ARID2, BAZ1B, BRD8, CHAF1A, CHD9, CPA4, EP400, FBXL10, GPX4, H2AFV, H2AFX, H2AFY2, H3F3B, HDAC4, HILS1, HIST1H4B, HIST1H4E, HIST1H4J, HIST1H4K, INOC1, JARID1B, JMJD1C, JMJD2D, MLL3, MYST1, MYST3, NAP1L1, NCOR1, PPARGC1A, RB1, RCBTB1, RERE, RING1, SAFB, SETD7, SETDB2, SETMAR, SIRT4, SIRT7, SRCAP, TAF6L, TLK1, TSPYL5 |
| GO:0045941~positive regulation of transcription | 2.84E-02 | APP, ARHGEF10L, ATOH1, BMP6, CCNE1, CNOT7, DDIT3, EPAS1, FOXF1, FOXH1, GLIS3, ILF3, ING1, INHBA, KLF13, KLF15, MEF2A, MKL2, MYST1, MYST3, NARG1, NEUROG3, NFATC2, NRIP1, NUP62, PIAS2, PPARGC1A, RB1, RXRA, SERTAD3, SIX4, SMAD1, SMAD3, SMAD5, SQSTM1, SRF, TEF, TFAP2B, THRAP3, TRERF1, UBB |
| GO:0046578~regulation of Ras protein signal transduction | 1.89E-02 | AKAP13, ARFGEF2, ARHGEF10L, ARHGEF15, BCR, DDEF1, EVI5L, FBXO8, FLJ10357, HRB, IQGAP1, ITSN1, KALRN, KIAA1975, MFN2, NET1, NUP62, PREX1, PSCD3, PSD4, SOS2, TBC1D2, TBC1D22A, TBC1D2B, TBC1D9, TIAM1, TRIO, VAV2 |
| GO:0007264~small GTPase mediated signal transduction | 1.79E-02 | AKAP13, ARFGEF2, ARHGAP1, ARHGAP29, ARHGEF10L, ARHGEF15, ARL10, ARL2, ARL5B, BCR, CDC42, CHP, DAB2IP, DDEF1, DOK3, EVI5L, FBXO8, FLJ10357, G3BP2, GAPVD1, GEM, GRB2, GTPBP4, HRB, IQGAP1, ITSN1, KALRN, KIAA1975, KRIT1, LOC286526, MFN2, NET1, NUP62, PREX1, PSCD3, PSD4, RAB18, RAB1A, RAB20, RAB27B, RAB35, RAB38, RAB39B, RAB6A, RAP2C, RASAL2, RGR, RHOV, RIN2, RIT1, SH2D3A, SOS2, TBC1D2, TBC1D22A, TBC1D2B, TBC1D9, TIAM1, TRIO, USP8, VAV2 |
